# Constructing internal audit quality evaluation index: evidence from listed companies in Jiangsu province, China

**DOI:** 10.1016/j.heliyon.2022.e10598

**Published:** 2022-09-13

**Authors:** Ren Kai, Kong Yusheng, Albert Henry Ntarmah, Chen Ti

**Affiliations:** School of Finance and Economics, Jiangsu University, Zhenjiang, PR China

**Keywords:** Internal audit quality evaluation index, Delphi method, Balanced scorecard, Systematic literature review, Analytical hierarchy process, Jiangsu province, China

## Abstract

Internal audit quality is the foundation for a company's survival and development across the world. As a result, global efforts have been made to develop a scientific and accurate evaluation index for internal audit quality. However, literature shows that existing internal audit quality evaluation indices have many flaws, such as a lack of systematic internal audit evaluation indicators, poor execution, and inability to identify priority areas. To address gaps in the literature, this study intended to construct an internal audit quality evaluation index utilizing the joint approaches of the Balanced Scorecard, Delphi Process, and Analytical Hierarchy Process. A systematic literature review, examination of companies' internal audit guidelines, Balanced Scorecard, and Delphi approaches resulted in a multilevel internal audit quality evaluation index with five dimensions (stakeholder satisfaction, stakeholder contribution, financial results, internal audit process, and learning and growth) and 36 indicators. The Analytical Hierarchy Process revealed that the most prioritized dimension is the internal audit process. The consistency ratio and evaluation feedback from the listed companies' internal auditors, management, and audit committee members revealed that the results are valid and reliable.

## 1 Introduction

Internal audit quality is the foundation for a organization's survival and development across the world. According to Boskou et al. (2019), internal audit quality (IAQ) is a critical component of an organization's sustainability since it assists the business in maintaining effective controls and avoiding fraud. Several publications (Ashbaugh-Skaife et al., 2008; Hoai et al., 2022) have shown that effective internal control may improve the quality of financial reporting. It is commonly acknowledged that IAQ is a critical component of successful internal control, on which financial reporting quality is also dependent. It helps the organization's operations since it is a separate activity that assists a corporation in achieving its goals by employing a well-organized approach to risk management, internal control, and governance efficiency and effectiveness (Stewart and Subramaniam, 2010; El Gharbaoui and Chraibi, 2021). Scholars from all around the world have discovered a link between IAQ and organizational performance (Hazaea et al., 2020; Kaawaase et al., 2021). Hence, IAQ is of interest to managers, shareholders, investors, auditors, and consequently international organizations and nations.

In line with the usefulness of the IAQ, there have been global efforts in establishing a scientific evaluation index for IAQ. International organizations and regulatory bodies such as the Financial Reporting Council (FRC) and the largest six audit firms in the United Kingdom; the Public Company Accounting Oversight Board (PCAOB) in the United States; the Chartered Accountants of Australia and New Zealand (CAANZ); the Accounting and Corporate Regulatory Authority (ACRA) in Singapore; and the International Organization of Securities Commissions (IOSCO) put forward indicators for evaluating IAQ. For instance, PCAOB published 28 indicators for evaluating audit quality while CAANZ published only nine indicators. The Committee of Sponsoring Organizations of the Treadway Commission (COSO) also published five components of a firm’s internal control system with 17 principles (COSO, 2013).

Despite growing interest in the evaluation of IAQ, researchers have not reached a consensus on a valid and reliable internal audit quality evaluation (IAQE) index. The evaluation indicators of IAQ developed by international organizations and regulatory bodies such as PCAOB and IOSCO resulted in inconsistent and ambiguous content, making the indicators redundant for many organizations (Harris and Williams, 2020). Trotman and Duncan (2018) revealed that different stakeholder groups in their study prioritized different IAQE dimensions making the indicators less reliable and inconsistent for achieving the intended purpose for many organizations. While Guizhen's (2019) index was limited to sewage treatment in China, Guilong’s (2013) focused on Communications Company. Consequently, the contents of their indicators are skewed to sewage and communications companies, which may be impracticable to other companies. Other indicators also ignored stakeholder and audit committees’ contributions (Souad et al., 2021) and financial results dimensions (Ganmei, 2015) in the development of IAQE. Hence, there is a quest for a reliable, valid, and scientific IAQE index system.

This research tries to construct an IAQE index. The study draws on literature review, multiple approaches, and the perspectives of listed companies in Jiangsu Province, China to build a scientific and reliable index system that addresses the flaws established in the literature and contributes to effective management controls and the organization’s survival. Over the years, China has seen significant growth in companies. Some local companies have expanded become multinational companies while many foreign companies have been established across many provinces in China. According to the National Bureau of Statistics of China (NBSC), the country is one of the fastest-growing in business (NBSC, 2019). As a result, developing the IAQE index can help to improve the quality of internal audits and the sustainability of businesses by enhancing financial reporting quality and reducing possible fraud.

The innovations of this study are as follows: (1) it constructs a new IAQE index. This study builds a multilevel IAQE index comprising five dimensions and 36 indicators. The index extends on existing indicators to capture stakeholder dimensions that have been missing in many studies. The index is comprehensive for measuring IAQ and improving organizational and institutional structures and processes needed by today’s businesses. By defining the main audit evaluation indicators according to different dimensions, the study guides IAQ improvement, quantifying subjective and objective indicators, and information for the comprehensive evaluation of audit projects. (2) It combines systematic literature review, the Balanced Scorecard (BSC), the Delphi technique, and Analytical Hierarchy Process (AHP) are used to build the IAQE index and as well as determining the weightings of the index system that are scientific, valid, and consistent. Combining these methodologies reveals the expertise and experiences of internal audit quality professionals and researchers, as well as the science of quantified measurement.

The following are the study's contributions: (1) it offers a complete and composite indicator for assessing IAQ. This study draws on the idea of BSC's balanced scorecard performance evaluation and builds an IAQE index system with 36 indicators grouped into five dimensions: stakeholder satisfaction; stakeholder contribution; financial results; auditing process; and learning and growth. The current study broadens the breadth and content of earlier studies (Harris and Williams, 2020; Gaosong and Leping, 2021) by including stakeholder dimensions that are essential to IAQE. (2) the study identified the internal audit process as the most priority area followed by stakeholder satisfaction. This implies that these areas need immediate attention within the IAQE index system. Thus, an evaluation of IAQ should aim at addressing the internal audit process, then stakeholder satisfaction before any other dimension.

The remainder of the work is arranged in the following sections: Section 2 deals with the literature review, while Section 3 discusses the methodology. Sections 4 and 5 focus on the results and discussion respectively. Finally, Section 6 presents conclusions and recommendations.

## 2 Literature review

The global need for a scientific and comprehensive index for the evaluation of IAQ has led to many international organizations and regulatory bodies investing in the development of IAQ evaluation indicators. These international bodies and organizations include CAANZ; PCAOB; Federal Audit Oversight Authority (FAOA) in Switzerland; the FRC and the six largest audit firms in the United Kingdom; and the United States Center for Audit Quality (US CAQ). However, researches show that IAQs outcomes bear some similarities but differ quite significantly throughout these organized bodies and countries worldwide (Federation of European Accountants, 2015). For instance, the indicator “training ours for auditing personnel” is common in FRC UK, PCAOB, US CAQ, IOSCO indicators. Similarly, “years of experience” and “technical resources support” are common among PCAOB and US CAQ indicators. On the other hand, “staff satisfaction” is available in only FRC UK and PCAOB indicators while “top-level involvement” is found in only PCAOB and US CAQ indicators. Furthermore, “use of new tools and approaches” is found in only PCAOB and CAANZ indicators. In general, while some bodies/organizations provide a set of up to 28 evaluation indicators for IAQs, others suggest less than ten evaluation indicators for IAQs (Federation of European Accountants, 2015). In addition, Harris and Williams (2020) established that many companies described some of the IAQ evaluation indicators developed by these regulatory bodies as infeasible, redundant, and ambiguous to implement.

At the domestic level, few authors from diverse fields attempted to develop the IAQE index. Guizhen (2019) constructed an internal audit evaluation system of sewage treatment in China. The content of the index system is mainly centered on sewage treatment. As a result, companies with diverse fields cannot adopt and implement such an index system. Using the BSC approach, Chaoli (2008) established that financial perspective, internal audit client, internal audit process, and learning and innovation perspectives form part of the IAQE Index. A major weakness in Chaoli’s study is that the author failed to consider stakeholder contributions such as the amount the company invested in internal auditing, the audit committee’s attention to corporate risks, the management level of reporting on internal audit requirements. Similarly, Guilong (2013) developed an internal audit performance evaluation (IAPE) model for a Communications Company. The author relied on coupling theory and a BSC to establish board satisfaction, management satisfaction, internal audit process, learning and growth, and financial dimensions as the focus of the IAPE index system. Judging from the index system, the author focused on board and management as the key stakeholders in IAPE but failed to consider audit committee members. Hence, the view of other stakeholders was not considered creating content defects in their index system. As argued by Souad et al. (2021) the stakeholder and audit committees’ contributions to the development of the IAQE index cannot be underestimated. Thus, an index system that considers all the key stakeholders is needed. After performing systematic literature review in China, Hazaea et al. (2021) concluded that internal audit literature in China has several weaknesses and there is more to be explored to fully understand and clarify various aspects of internal audit in China.

Another group of scholars concentrated on understanding the dimensions of the IAQE index. Harris and Williams (2020) examined the dimensions of the evaluation index for IAQ of Non-Fig Four audit firms in the USA. The authors used the USA Public Company Accounting Oversight Board (PCAOB) framework to group the evaluation index of IAQ into three main areas: audit professionals, audit process, and audit results. They concluded that timely reporting of internal audit results, the knowledge, specialized skills, and experience of auditor personnel should be given high recognition. However, the authors failed to consider the importance of stakeholder dimensions in constructing the IAQE index. As pointed out by Azzali and Mazza (2018), stakeholders play a key role in the evaluation of IAQ. Hence, ignoring stakeholder dimensions may create defects in the index system. Trotman and Duncan (2018) investigated the evaluation index for IAQ from the perspectives of multi-stakeholders in major accounting firms. The authors constructed five-dimensional quality indicators - input, process, context, output, and outcome. Nevertheless, the authors could not differentiate between some of the dimensions. Some items within the output and outcome dimensions overlapped. Hence, different stakeholder groups in the study prioritized different quality dimensions. In this case, such an index may not be reliable to implement in different organizations. Similarly, the work of Ganmei (2015) neglected the financial results dimension where fraud detection is key.

In line with the weaknesses pointed out, Yang et al. (2017) described existing evaluation indicators for IAQ as having many defects such as lack of systematic internal audit evaluation indicators, poor execution, and failure to identify priorities areas. These limitations usually arise due to over-reliance on the approved internal regulations of specific organizations, Code of Ethics, established traditions, and practical experience (Nedyalkova, 2020). Similarly, Zhu et al. (2021) used survey data, principal component analysis, and probabilistic hesitant fuzzy elements to study evaluation information of internal control audits of some universities in China. The authors concluded that evaluation systems for IAQs are not perfect and the professional knowledge and skills of internal auditors are not adaptable. Based on the gaps identified in the literature, this study seeks to construct an IAQE index system based on systematic literature review, local and international standards of internal audit quality as well as an existing evaluation framework for IAQ used by multiple companies, and BSC perspectives. The study will further evaluate the index using the Delphi process and AHP approaches.

## 3 Methodology

### 3.1 Study area

**Figure 1:**
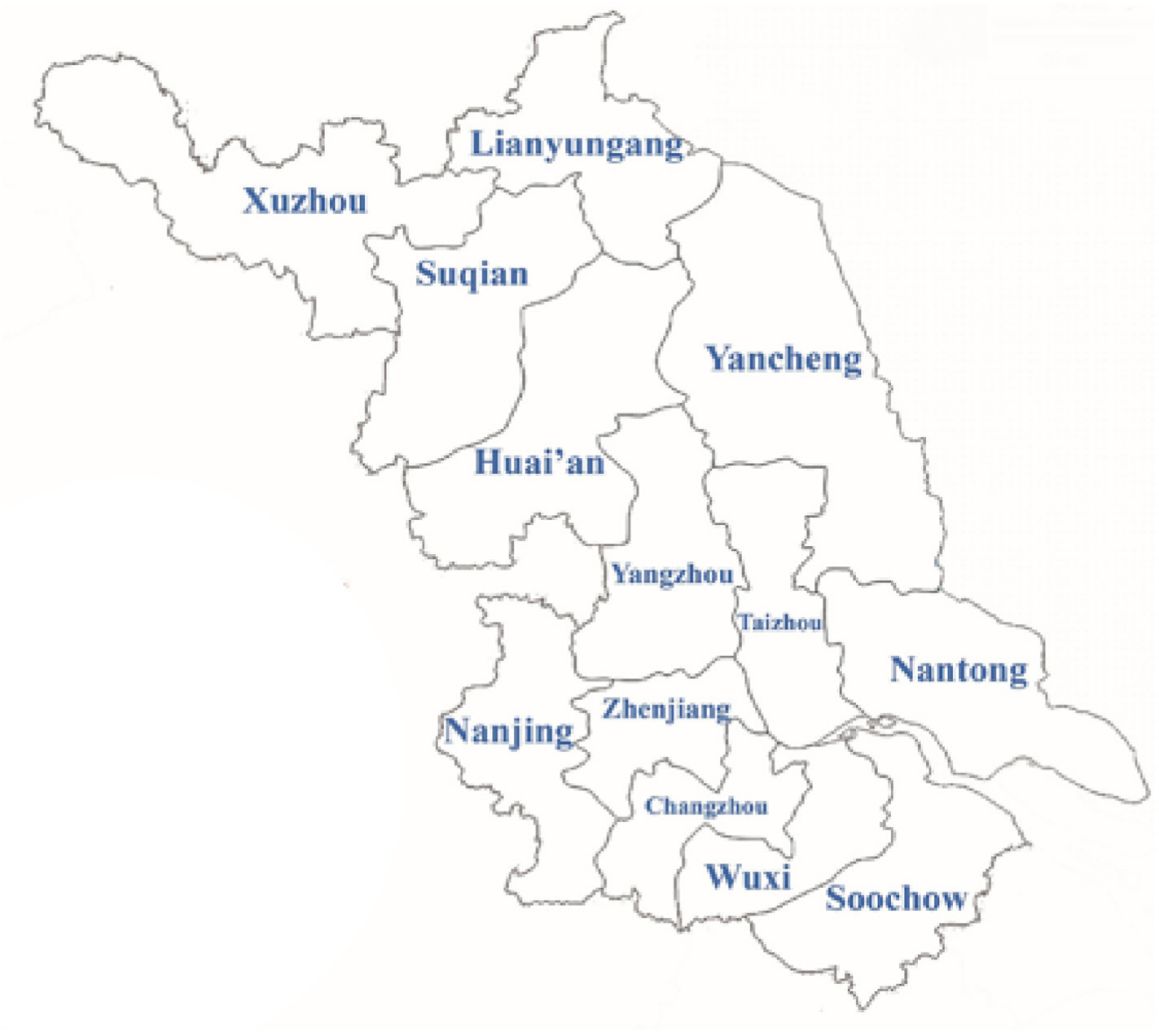
Map of jiangsu province.

Jiangsu Province is located on the eastern-central coastline of China. It shares a boundary with Shandong Province in the north, the Yellow Sea in the east, Zhejiang Province in the south, and Shanghai Province in the west. It is the fifth most populous province in China with a population of 80.70 million (NBSC, 2019). It is 102,600 square kilometers in area with 13 cities (see Figure 1) It is very flat, with plains accounting for 68 percent of the total area and water contributing for 18 percent. It has a coastline of nearly 1,000 km, and the Yangtze River flows through the province's southern section, making it an important component of the lower Yangtze River Basin. The NBSC (2019) data shows that Jiangsu Province contributed 9.96 trillion Yuan to China's economic growth, ranking second among Chinese provinces. Its secondary industry contributed 4.23 trillion Yuan to China's economic growth, making it the country's second-largest enterprise contributor (NBSC, 2019). It has the second-highest international trade in goods of foreign-invested enterprises in China (USD 375 billion). It is also the second-largest investment by foreign-invested enterprises in China, totaling USD 1.17 trillion (NBSC, 2019). There are 428 listed firms with their headquarters in Jiangsu Province, China (Statista, 2021). These firms are operating across the provinces in China. This research seeks to build an IAQE index using Jiangsu province as a study region since the province offers a strong foundation point for constructing a complete and realistic IAQE index.

### 3.2 BSC approach

According to the Institute of Internal Auditors, “internal auditing is an independent, objective assurance and consulting activity designed to add value and improve an organization's operations” (IIA, 2022). It assists a company in achieving its goals by applying a systematic, disciplined approach to evaluating and improving the efficacy of risk management, control, and governance systems. In line with this, the development of IAQE index should be completed within a multi-criteria decision making (MCDM) framework. The BSC is one of the MCDM and powerful management accounting instrument for organizational change and as well as an effective performance monitoring system. It converts a company's mission, objectives, and goals into performance metrics. The first BSC had four perspectives: stakeholder, financial, internal process, and learning and growth (Wu, 2012; Rababah, 2014). However, recent studies have provided a modified the initial BSC framework into five perspectives (Chung et al., 2016). According to BSC, a single performance indicator cannot accurately reflect the performance of a complex organization (Nasiripour et al., 2012). The BSC is increasingly being used to assess and report on the quality of internal audits. Indicators are placed in diverse viewpoints in this strategy, which gives a balanced assessment of performance and supports strategic decisions at firms (Acuña-Carvajal et al., 2019). The BSC has been widely employed by a variety of organizations throughout the world to assist companies in converting their purpose and strategy into indicators that help the analysis of outcomes and decision making following the management control system (Acua-Carvajal et al., 2019).

To extract relevant indicators for the construction of the IAQE index, we first formed a committee of six Chinese and foreign scholars (see Table 1 for the demographics of the scholars) in the field of corporate governance and internal auditing who are working in our department. The scholars are experienced researchers who are consultants to domestic and foreign companies. Second, the committee conducted a preliminary review of IAQE systems and related issues from selected companies in Jiangsu Province. The Committee first contacted 35 companies in the province. There were 27 companies that agreed to take part in the study. However, three of them could not go through the entire process. Hence, the preliminary information was limited to 24 companies. These companies are operating with both Chinese and international standards for internal auditing and have branches in many provinces in China. The preliminary information focused on reviewing the selected companies’ internal auditing guidelines, checklist, and code of ethics as well as interviewing audit committee members about possible issues regarding IAQE. Relevant indicators were extracted during the process. Forth, the committee performed a systematic literature review on the topic (using scholarly databases including the Global Audit Information Network, Web of Science, Science Direct, Scopus, and Google scholar). Journal articles, international audit standard documents, books, and conference papers were downloaded and reviewed. Relevant indicators identified in the literature were extracted. Finally, all the indicators extracted from the preliminary information and systematic literature review were merged. By consensus, the committee deleted indicators that were found to be repeated to avoid duplication. The pre-selection of indicators through systematic literature review thrives on the theoretical foundations of Fatima and Elbanna (2020) and Chung et al. (2016) who incorporated literature review in BSC application.

**Table 1:** Demographics of Chinese and foreign scholars in the field of corporate governance and internal auditing.

#	Sex	Age in Years	Education	Rank	Experience in Years
1	Female	41	PhD	Associate Professor	17
2	Male	46	PhD	Professor	20
3	Male	47	PhD	Professor	24
4	Female	52	PhD	Professor	27
5	Male	56	PhD	Professor	27
6	Male	59	PhD	Professor	32

Using the modified BSC framework, the committee developed the initial IAQE index by classifying the indicators into five BSC perspectives – stakeholder satisfaction, stakeholder contributions, financial results, internal audit process, and learning and growth perspectives. Thus, the original stakeholder perspective has been decomposed into stakeholder satisfaction and stakeholder contributions perspectives. According to the experts, proper implementation requires perspectives to be less ambiguous. Hence, adopting the five BSC perspectives (Bhattacharya et al. 2014; Chung et al., 2016) will give better clarity and proper implementation than the initial four BSC perspectives.

### 3.3 Delphi method (DM)

The DM is a group consensus technique that employs a systematic study of research, stakeholder opinions, and the judgment of experts in an area to establish an agreement after numerous rounds of discussions (Monguet et al., 2017). The DM is beneficial when evidence is insufficient or limited: it depends on group members' “collective intelligence” to create better outcomes than any individual in the group could achieve on his or her own, resulting in greater content validity and reliability (Sullivan, 2011; Zielskea and Held, 2021). In this work, we used the DM to reach an agreement on an adequate and trustworthy IAQE index comprised of major criteria and particular indicators. DM has been demonstrated to be the most beneficial, dependable, and acceptable consensus-building methodology across different disciplines (Yusheng and Ntarmah 2021). The use of DM necessitates professional consensus on the index's contents through numerous rounds of discussions and screening (Yusheng and Ntarmah, 2021).

In this study, we first invited 45 experts/scholars in the field to complete the Delphi survey through electronic mail. The overview and purpose of the survey as well as the processes the DM with follow and timelines were outlined in the e-mail. We received feedback from 37 of the participants (See Table 2 for the demographics of the participants involved in the study) agreeing to be part of this study. The DM participants were 12 internal audit experts/auditors, 11 audit committee members from the selected listed companies in Jiangsu Province, China, and 14 scholars in the field of corporate governance and internal audit from academia. The participants rated the importance of the indicators on a nine-point scale (1–9) ranging from one (1) as not important to nine (9) as very important. Second, criteria for item inclusion were created. The participants set the screening criterion for the inclusion of an indicator in the index at the widely suggested geometric mean of 7.54 (Chen et al., 2018). Third, we distributed to the participants the 43 pre-selected indicators and scoring criteria. Participants were asked to completely assess the indications, assign a score to each indicator based on the scoring criteria, and provide feedback on their score. After two rounds of expert consultations, a consensus on desired indicators was reached. The response from the first round of surveys was utilized to create the second round. After the second round, indicators that fulfilled the criterion were included in the index system.

**Table 2:** Demographics of the participants involved in delphi method.

#	Sex	Age in Years	Education/Qualification	Rank	Experience in Years
1	Female	40	Chartered Accountant	Audit Committee Members	15
2	Male	35	Masters	Internal Audit Experts/Auditors	9
3	Male	38	Chartered Accountant	Audit Committee Members	12
4	Female	40	PhD	Corporate Governance (Internal Audit)	14
5	Female	49	Masters	Audit Committee Members	23
6	Male	34	Chartered Accountant	Internal Audit Experts/Auditors	7
7	Male	39	PhD	Corporate Governance	12
8	Male	43	PhD	Corporate Governance (Internal Audit)	16
9	Female	46	Chartered Accountant	Internal Audit Experts/Auditors	19
10	Female	38	Masters	Internal Audit Experts/Auditors	10
11	Male	38	PhD	Corporate Governance	10
12	Male	41	Masters	Audit Committee Members	13
13	Male	35	Chartered Accountant	Audit Committee Members	6
14	Male	36	Masters	Audit Committee Members	7
15	Male	38	Chartered Accountant	Internal Audit Experts/Auditors	9
16	Female	44	PhD	Corporate Governance	15
17	Female	48	PhD	Internal Audit	19
18	Male	52	PhD	Internal Audit	23
19	Male	42	PhD	Corporate Governance	12
20	Female	44	Chartered Accountant	Internal Audit Experts/Auditors	14
21	Female	48	Chartered Accountant	Internal Audit Experts/Auditors	18
22	Female	49	Chartered Accountant	Audit Committee Members	19
23	Male	54	PhD	Corporate Governance (Internal Audit)	24
24	Male	55	PhD	Internal Audit	25
25	Male	56	Masters	Audit Committee Members	26
26	Male	57	Masters	Audit Committee Members	27
27	Male	39	Chartered Accountant	Internal Audit Experts/Auditors	8
28	Male	41	Masters	Internal Audit Experts/Auditors	10
29	Female	57	PhD	Internal Audit	26
30	Male	59	PhD	Corporate Governance (Internal Audit)	28
31	Female	48	PhD	Corporate Governance (Internal Audit)	16
32	Male	56	PhD	Corporate Governance	24
33	Male	48	Chartered Accountant	Audit Committee Members	15
34	Male	52	Masters	Audit Committee Members	19
35	Female	56	Chartered Accountant	Internal Audit Experts/Auditors	22
36	Male	52	Masters	Internal Audit Experts/Auditors	17
37	Male	50	Chartered Accountant	Internal Audit Experts/Auditors	14

### 3.4 Analytical Hierarchy Process (AHP)

AHP is a structured and MCDM technique for problem-solving that is used to tackle difficult decision issues (Leal, 2020). It has a multi-level hierarchical structure that includes objectives, criteria, sub-criteria, and choices. It provides a wide-ranging and logical structure for developing a decision issue and evaluating potential solutions. The approach was invented by Saaty (1980). It has since been employed in a wide range of scientific areas (Qin et al. 2021). In many research in various fields, AHP is regarded as the most successful and widely utilized approach of MCDM (Leal, 2020; Yusheng and Ntarmah 2021). It enables the incorporation of quantitative and qualitative aspects into decision-making processes (Akbar et al. 2020). The key imperative measures of the AHP technique are generating the assessment problem and building a hierarchy. Following the creation of the hierarchy, the decision-maker can rank the components to evaluate their relative worth at each level of the hierarchy. In terms of weight, elements are examined and compared pairwise at each level (Hussain et al., 2021). The following stages are often used in the implementation of AHP: (1) Build a hierarchy model; (2) Construct pairwise comparison matrices; (3) Use numerical analysis to determine the level weight factors and acquire the eigenvector, and (4) Perform a consistency test.

#### 3.4.1 Hierarchy model construction

The hierarchy model creation is described as the foundation of the AHP approach as the first stage in the AHP application. It takes a top-down strategy, starting with the highest level (objective) and working down to lower-level decision variables (Waris et al., 2019). A three-level hierarchy model is developed in this study. From top to bottom, IAQE is hierarchized, and an index system model with a separate hierarchical structure is built. The top level reflects the comprehensive evaluation's aim (IAQE); the second level is the major criterion for assessing IAQE, and the third level is the sub-criteria (specific indicators). When a decision problem is organized as a hierarchy, the complicated problem is set out plainly.

#### 3.4.2 Pairwise comparison

The relative importance of the main criteria and sub-criteria are compared in pairs at this stage. Throughout this procedure, the items in each set in the hierarchy are compared to their corresponding group members (Munizu and Riyadi 2021). Professionals in the field are frequently asked to assess the relative importance of issues using the widely used Saaty's nine-point scale (see Table 3) (Upreti et al., 2019). The scale goes from one to nine, with one indicating “equal importance of both indicators” and nine indicating “one indicator is much more important than the other.” As a consequence, the experts provide a numerical value to the judgment based on their pick. The pairwise judgments are recorded in a decision matrix. A comparison matrix is represented algebraically in Eq. (1).

(1) $$A=\left[\begin{array}{cccc} 1 & a_{12} & ... & a_{1n}\\ 1/a_{12} & 1 & ... & a_{2n}\\ \vdots & \vdots & \cdots & \vdots \\ 1/a_{1n} & 1/a_{2n} & \cdots & 1 \end{array}\right]$$

**Table 3:** Nine-point system.

Scaling	Meaning
1	Both indicators are equally important
3	One indicator is slightly more important than the other
5	One indicator is more important than another
7	One indicator is significantly more important than another
9	One indicator is immensely more important than another
2, 4, 6, 8	Between the above two adjacent situations
The reciprocal of the above	Compare the two indicators in turn

The “A” matrix is a n × n matrix that represents pairwise comparison or the relative importance of alternatives, where n is the number of items examined. The matrix “A” has the following properties:

$a_{ii}=1\Leftrightarrow i=j,$ signifying that an item judged against itself is one (1). $a_{ji}=\frac{1}{a_{ij}},$ representing a reciprocal matrix of each entry $a_{ij}$. The entries $a_{ij}$ are the relative judgments between the two alternatives i and j in a manner in which the ith row matches to the jth column of matrix “A.”

#### 3.4.3 Weight determination

The third stage in the AHP application is to calculate the relative weights of each of the hierarchy model's primary and sub-criteria. The eigenvector method is commonly used to determine this (Ekmekciolu et al. 2021). The eigenvector method is based on matrix theory. This approach is utilized since it is acceptable, trustworthy, and useful in establishing the weights of many indices (Ekmekciolu et al. 2021). To calculate the weights, the eigenvector approach compares the normalized eigenvalue to the primary eigenvalue (Waris et al. 2019). The relative weights of criteria can be written in a matrix form in Eq. (2) by following the pairwise comparison matrix “A” in Eq. (1)

(2) $$A=\left[\begin{array}{llll} w_{1}/w_{1} & w_{1}/w_{2} & ... & w_{1}/w_{n}\\ w_{2}/w_{1} & w_{2}/w_{2} & ... & w_{2}/w_{n}\\ \vdots & \vdots & ... & \vdots \\ w_{n}/w_{1} & w_{n}/w_{2} & ... & w_{n}/w_{n} \end{array}\right]$$

(3) $$w=\left(w_{1},w_{2},w_{3}...w_{n}\right)$$

Where “w” represents the eigenvector and a column matrix. The eigenvector in Eq. (3) is constructed using geometric mean approach (Waris et al. 2019).

#### 3.4.4 Consistency test

Sometimes, there are inconsistencies in the experts' judgments during pairwise comparisons. In the presence of inconsistencies, the matrix cannot be accepted and the judgments must be amended (Chen et al. 2018). The consistency test can help with this. The consistency ratio (CR) is a tool for determining the consistency of optimal decision-making (Shen et al. 2019). This helps to determine whether the judgment is free from logical errors. The CR is represented in Eq. (4) as:

(4) $$CR=\;\frac{CI}{RI}\leq 0.1$$

Where $CI=\;\frac{\lambda_{\max}-n}{n-1}$ CR denotes consistency ratio, CI indicates consistency index, and RI (random index) stands for the randomly produced reciprocal matrix of the consistency indicators from Saaty's 9-point scale. The largest eigenvalue is represented by λ_max_, while the matrix's rank is represented by “n.” To be regarded as acceptable, the CR should be less than or equal to 0.10 (CR 0.10).

### 3.5 Evaluating the performance of IAQE index among listed companies

After determining the index weights of the IAQE, it is important to evaluate the performance of the index based on the perspectives of selected listed companies. With the help of research assistants, we conducted an online survey from March 2021 to June 2021 to evaluate the index. Using an online survey, we distributed 528 questionnaires (see Appendix for questionnaire) to the internal auditors, management, and audit committee members of 176 listed companies in Jiangsu Province, China. Participants were asked to rate the indicators on a 10 points scale ranging from 1 less important to 10 very important. There were 364 fully completed and returned questionnaires. Based on the literature, the individual score for an indicator can be computed:

(5) Z=W×I

Where Z is the computed score for an indicator, W is the weight of the indicator, and I is the average rating of an indicator. Eq. (5) can be modified to represent the overall score (see Eq. 6) of IAQE indicators as:

(6) ∑Z=∑(W×I)

**Figure 2:**
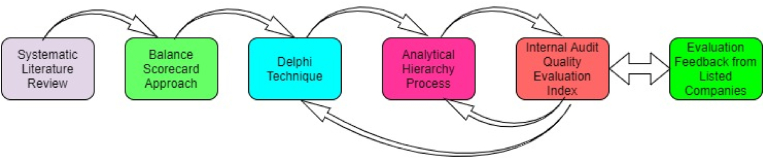
Research process.

Where ∑ represents the summation of the score. Other variables are defined in Eq. (5). Figure 2 represents the research process.

### 3.6 Ethical considerations and quality control

We explained the study's objectives and methods to the participants and they volunteered to take part in this research. The Jiangsu University's Research Institute of Financing and Accounting and the Ethics Committee approved the protocol for this study. We also requested consent from the firms engaged in the study through the internal audit agency. Furthermore, we wanted participants to have the freedom to choose whether or not to engage in this study, as well as the capacity to decline or withdraw their commitment to participate at any time without penalty. We ensured the participants of their confidentiality and anonymity. To protect the participants' privacy and confidentiality in the DM and AHP procedures, we assigned each questionnaire a unique code. In addition, no records or traces of any participants were found in the summary feedback of the reports supplied to participants.

We utilized a strict method to ensure the quality of this study. Under the supervision of our research department, we selected individuals based on a range of variables. For example, while selecting participants for the DM and AHP approaches, we considered their educational background, in-depth knowledge, research experience, and interest in this topic. In addition, we examined people who reside in the province and are familiar with rural development in the area. We described the study's aim as well as the procedures that would be followed. We also defined the duties of the participants and the grading criteria. Finally, participants were made aware of the most recent research in the subject before expert consultations for the first and second DM rounds, as well as the AHP application.

## 4 Results

### 4.1 BSC results

A systematic literature review on the topic and existing IAQE indicators combined with experts’ review of companies’ internal audit quality framework, 52 indicators were initially extracted. Based on experts’ consensus, some indicators were merged with other indicators while others were removed due to their little relevance to the index system. The process reduced the indicators from 52 to 43. The experts classified the 43 indicators into five BSC perspectives – Stakeholder satisfaction (9 indicators), Stakeholder Contribution (9 indicators), Financial Results (8 indicators), Internal Audit Process (9 indicators), and learning and growth (8 indicators) perspectives. The final indicators under each of the five BSC perspectives were further assessed using DM and AHP approaches.

### 4.2 Delphi method results

**Figure 3:**
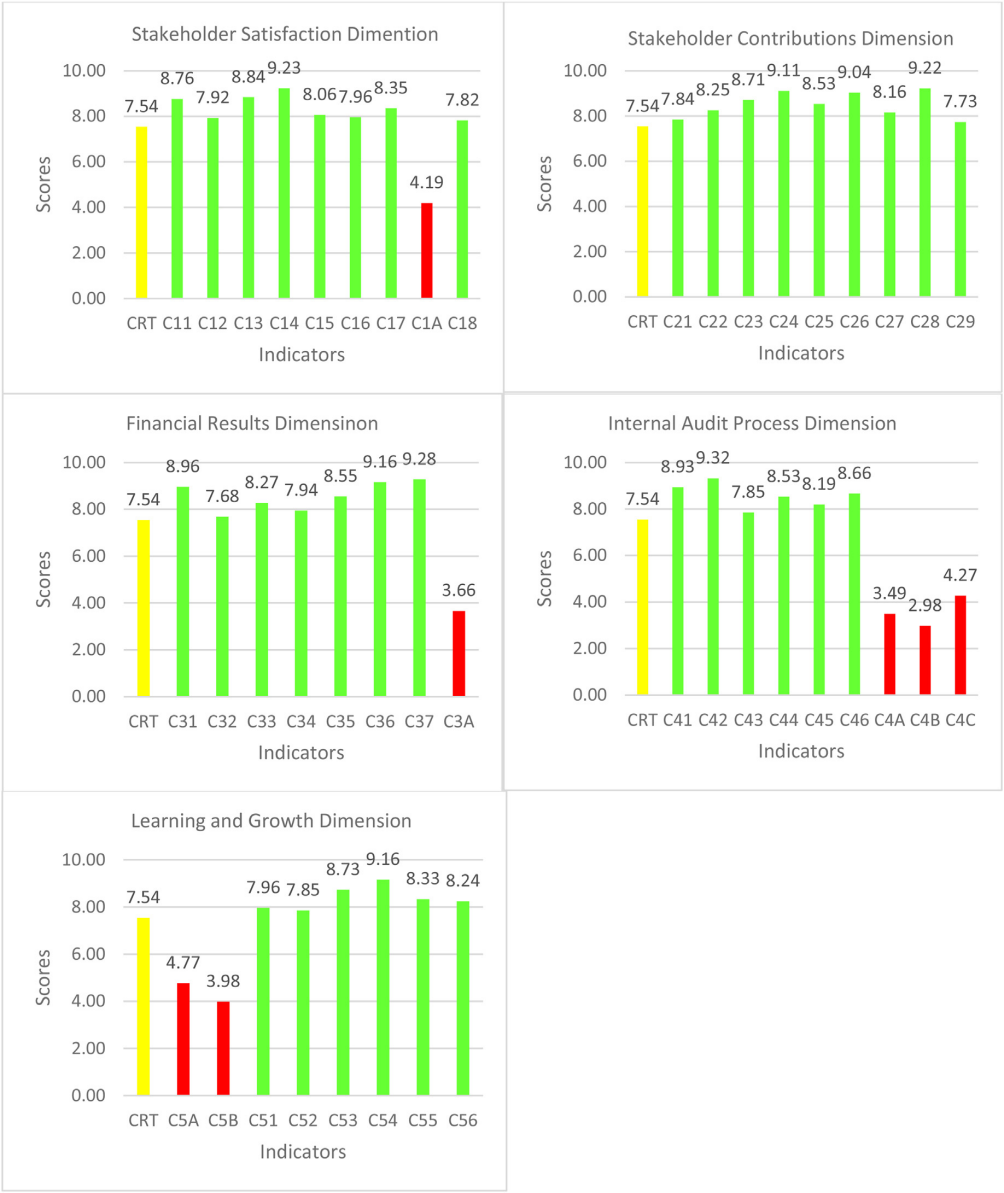
Dm results for IAQE dimensions and indicators.

We used the 43 indicators classified into five BSC perspectives to develop an initial questionnaire for the Delphi process. The analysis of 37 experts who completed the questionnaire for the Delphi process is presented in Figure 3 and Table 4. In Figure 3, the criterion value (CRT) is 7.54, represented as yellow, green represents suitable indicators for inclusion in the IAQE index system, and red represents unsuitable indicators recommended for exclusion in the IAQE index system.

**Table 4:** IAQE index.

Target Layer	Code	First level (Dimensions)	Code	Second Level (Indicators)
Internal Audit Quality Evaluation (IAQE) Index	C_1_	Stakeholder satisfaction	C_11_	Management's functional positioning of internal audit
			C_12_	Satisfaction degree of State-owned Assets Supervision and Administration Commission with internal audit
			C_13_	Audit committee’s satisfaction with internal audit
			C_14_	The degree of management's acceptance of internal audit conclusions
			C_15_	Satisfaction degree of the auditee with the internal audit
			C_16_	Number of complaints received by the internal audit department
			C_17_	Satisfaction of the external audit agency with the internal audit provided by the internal audit department
			C_18_	The extent to which internal audit results are utilized by the external audit structure
	C_2_	Stakeholder contribution	C_21_	State-owned Assets Supervision and Administration Commission's control of internal audit functions
			C_22_	The level of management's implementation of the internal audit requirements of the State-owned Assets Supervision and Administration Commission
			C_23_	Number of audits requested by management
			C_24_	Management level of reporting on internal audit requirements
			C_25_	Frequency of direct meetings between the head of the internal audit and the audit committee
			C_26_	The audit committee's attention to corporate risks
			C_27_	How much does the company invest in internal auditing?
			C_28_	The proportion of companies implementing audit recommendations
			C_29_	Frequency of communication between external and internal auditors
	C_3_	Financial Results	C_31_	The internal audit found that the company's expenses were reduced
			C_32_	The rate of difference between internal audit fees and budget
			C_33_	What is the amount of fraud discovered by the internal audit?
			C_34_	Use internal audit to save external audit fees
			C_35_	The ratio of internal audit value added to internal audit cost
			C_36_	Number of major audit findings and recommendations
			C_37_	Audit recommendations are adopted and implemented
	C_4_	Internal audit process	C_41_	Importance of audit matters
			C_42_	The proportion of achieving internal audit objectives
			C_43_	The extent to which information technology is used in internal auditing
			C_44_	The degree of continuous improvement of the internal audit process
			C_45_	Timeliness of audit reports
			C_46_	Degree of perfection of internal audit quality assessment results
	C_5_	Learning and growth	C_51_	The average age of auditors
			C_52_	Average years of audit experience
			C_53_	Education level of auditors
			C_54_	Average hours of annual professional reeducation of auditors
			C_55_	Auditor's ability to use information technology
			C_56_	The proportion of employees with professional certification

At the end of the Delphi process, the experts’ recommended seven indicators to be deleted from the index system (see Figure 3 for red indicators) because they did not meet the threshold. Indicator C1A “staff satisfaction with internal audit” belonging to the stakeholder satisfaction dimension had the lowest consensus mean value of 4.19. Indicator C3A “inconsistencies in financial reporting” belonging to the financial results dimension had consensus mean values of 3.66. Similarly, within the internal audit process dimension, indicators C4A “focus areas of internal auditing”, C4B “regular audit reporting to management”, and C4C “quality of internal audit” had consensus mean values of 3.49, 2.98, and 4.27 respectively. Finally, the experts reported mean values of 4.77 and 3.98 for indicators C5A “training hours per audit personnel” and C5B “years of experience” suggesting that should be excluded from the learning and growth dimension. These seven indicators’ mean values were substantially below the criteria value, indicating that they did not reach the minimal level for inclusion as IAQE indicators. The experts' main justification for ignoring these indicators is that some of these items do not make any meaningful contribution (C1A and C4A) to the index system while others (C3A, C4B, C4C, C5A, and C5B) are already captured under other critical indicators within the index system making them noisy indicators.

After the experts’ consultations and scoring based on the inclusion criteria (7.54 threshold), 36 indicators (see Table 4 for approved indicators and their codes and Appendix for graphical illustration of the IAQE index) were retained as suitable for inclusion in the IAQE index system. The final 36 indicators approved based on BSC classifications and experts’ consensus was: eight indicators for stakeholder satisfaction, nine indicators for stakeholder contributions, seven indicators for financial results, six indicators for the internal audit process, and six indicators for learning and growth dimensions.

### 4.3 AHP results

At this point, analysis of the 46 completed AHP questionnaires (15 experts/auditors, 18 academic scholars, and 13 internal audit committee members) was performed. Based on the pairwise comparison and judgment matrix of 46 completed questionnaires, the weightings and consistency results were generated. The relevance of experts completing the questionnaire is emphasized in the AHP application, but a big sample size is not required (Kamaruzzaman et al. 2018). The suggested sample size for completing the AHP questionnaire is between 2 and 20 participants since greater sample sizes may result in arbitrary replies and substantial inconsistency (Kamaruzzaman et al. 2018). As a result, the AHP sample drawn from the three groups of participants falls within the acceptable sample size. Following the processes specified in the methodology, we offer the findings from the AHP questionnaire. Because the CR value is less than 0.1, there was no amendment made in the experts' judgment as their results remain acceptable and devoid of logical mistakes.

#### 4.3.1 Hierarchy model construction

**Figure 4:**
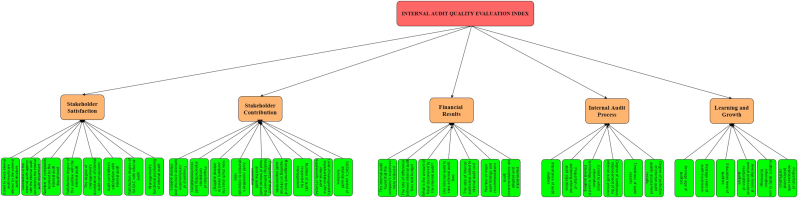
Three level hierarchy model construction of IAQE index.

Based on the DM results, we constructed a three-level hierarchy model of the IAQE index as represented in Figure 4. The highest level of the hierarchy represented as goal/target is the IAQE index. The second level is the dimensions of the IAQE index. These dimensions are stakeholder satisfaction, stakeholder contributions, financial results, internal audit process, and learning and growth. The last level represents the specific indicators that measure each of the five dimensions. Stakeholder contributions had the highest number of indicators followed by stakeholder satisfaction and financial results while internal audit process and learning and growth both had six indicators each being the least.

#### 4.3.2 AHP results of main criteria

Based on the analysis of experts’ judgment through a pairwise comparison matrix, the weightings and consistency test results of the dimensions and indicators are generated. The results in Table 5 show that the experts regard all five dimensions as important in the IAQE index. However, C_4_ had the highest weightings by all three groups of experts, suggesting that the internal audit process dimension is the most crucial in the IAQE index system. Internal Auditors/experts and academic scholars believe that C_1_ is the next important priority followed by C_3_ but audit committee members think otherwise. Although this outcome diverges slightly among the participants, they agree that the first three most prioritized dimensions implementing the IAQE index are C_4_, C_1_, and C_3_. C_5_ was ranked as the fifth suggesting that it should be the least to be considered in order of importance. It is worth stating that ranking C_5_ fifth does not mean that it is not important, however, the ranking signifies the order in which evaluation of IAQ should be implemented.

**Table 5:** AHP results on first level indices – main dimensions.

Main Dimensions (C)	Experts/Auditors		Academic Scholars		Audit Committee Members	
	W	Rank	W	Rank	W	Rank
C_1_	0.251	2	0.267	2	0.194	3
C_2_	0.133	4	0.117	4	0.133	4
C_3_	0.173	3	0.208	3	0.234	2
C_4_	0.353	1	0.329	1	0.359	1
C_5_	0.090	5	0.078	5	0.079	5
Consistency Checks	**5.30** (0.07)		**5.28** (0.06)		**5.29** (0.06)	

Bolded values represent λmax. CR values are in parenthesis ().

#### 4.3.3 AHP results for specific indicators

In terms of stakeholder satisfaction, the results in Table 6 show that C_14_ had the highest weight suggesting that all the respondent groups agreed that management's acceptance of internal audit conclusions is the most crucial indicator among eight stakeholder satisfaction indicators. On the other hand, auditors and academic scholars believe that C_16_ had the lowest weightings suggesting that the number of complaints internal audit departments receive is the least recognized indicator within the stakeholder satisfaction indicators. All the expert groups recognized C_13_ and C_11_ as the second and third priority indicators among stakeholder satisfaction indicators. Although the expert groups approved the importance of C_15_ and C_17_ within stakeholder satisfaction indicators, their recognition for fourth and fifth priority indicators diverge slightly (see Table 6). This is similar to the recognitions given to the seventh and eighth priority indicators.

**Table 6:** AHP results for stakeholder satisfaction indicators.

	Experts/Auditors		Academic Scholars		Audit Commitment Members	
C_1_	W	Rank	W	Rank	W	Rank
C_11_	0.166	3	0.169	3	0.160	3
C_12_	0.069	6	0.071	6	0.075	7
C_13_	0.172	2	0.180	2	0.168	2
C_14_	0.265	1	0.251	1	0.255	1
C_15_	0.111	5	0.112	4	0.097	5
C_16_	0.035	8	0.051	8	0.078	6
C_17_	0.115	4	0.106	5	0.108	4
C_18_	0.067	7	0.060	7	0.060	8
Consistency Checks	**8.21** (0.03)		**8.35** (0.04)		**8.54** (0.05)	

Bolded values represent λmax. CR values are in parenthesis ().

Concerning the stakeholder contribution dimension, Table 7 shows that C_28_ had the highest weight suggesting that the “proportion of companies implementing audit recommendation” is the most priority indicator among stakeholder contribution indicators. This is followed by C_24_ “management level of reporting on internal audit requirements” and C_26_ “the audit committee's attention to corporate risks”. However, C_29_ had the lowest weightings suggesting that “frequency of communication between external and internal auditors” is the least recognized indicator within the stakeholder contribution dimension.

**Table 7:** AHP results for stakeholder contribution indicators.

	Experts/Auditors		Academic Scholars		Audit Commitment Members	
C_2_	W	Rank	W	Rank	W	Rank
C_21_	0.041	8	0.056	8	0.058	8
C_22_	0.072	6	0.080	6	0.085	6
C_23_	0.098	4	0.097	4	0.099	4
C_24_	0.194	2	0.179	3	0.170	3
C_25_	0.089	5	0.093	5	0.089	5
C_26_	0.193	3	0.188	2	0.176	2
C_27_	0.057	7	0.064	7	0.068	7
C_28_	0.216	1	0.198	1	0.206	1
C_29_	0.039	9	0.045	9	0.050	9
Consistency Checks	**9.36** (0.03)		**9.84** (0.07)		**9.70** (0.06)	

Bolded values represent λmax. CR values are in parenthesis ().

The experts’ judgment on the financial results indicators shows that C_37_ “adoption and implementation of audit recommendations” is the most crucial indicator (see Table 8). Other indicators C_31_ and C_36_ received relatively high weightings indicating that audit findings and recommendations are key to promoting financial results. In contrast, “the rate of difference between internal audit fees and budget” (C_32_) had the lowest weightings among the financial results indicators. The findings depict unanimous agreement by the three groups of respondents in terms of order of priority of implementing financial results indicators.

**Table 8:** AHP results on financial results indicators.

	Experts/Auditors		Academic Scholars		Audit Commitment Members	
C_3_	W	Rank	W	Rank	W	Rank
C_31_	0.144	3	0.150	3	0.137	3
C_32_	0.037	7	0.062	7	0.056	7
C_33_	0.085	5	0.089	5	0.099	5
C_34_	0.079	6	0.083	6	0.076	6
C_35_	0.095	4	0.100	4	0.107	4
C_36_	0.251	2	0.231	2	0.240	2
C_37_	0.310	1	0.284	1	0.285	1
Consistency Checks	**7.19** (0.02)		**7.61** (0.08)		**7.56** (0.07)	

Bolded values represent λmax. CR values are in parenthesis ().

Concerning Table 9, the respondents rated C_42_ as the most priority indicator among the internal audit process indicators because it focuses on achieving internal audit objectives. Respectively, C41 and C46 were rated as second and third priority indicators in the internal audit process dimension. The rest of the indicators received considerably high weightings suggesting that all the items have some degree of importance.

**Table 9:** AHP results for internal audit process indicators.

	Experts/Auditors		Academic Scholars		Audit Commitment Members	
C_4_	W	Rank	W	Rank	W	Rank
C_41_	0.154	2	0.168	2	0.169	2
C_42_	0.374	1	0.343	1	0.338	1
C_43_	0.072	6	0.078	6	0.114	6
C_44_	0.139	4	0.141	4	0.138	4
C_45_	0.111	5	0.115	5	0.089	5
C_46_	0.151	3	0.155	3	0.152	3
Consistency Checks	**6.20** (0.03)		**6.23** (0.04)		**6.38** (0.06)	

Bolded values represent λmax. CR values are in parenthesis ().

The CR results signify valid and acceptable results for the internal audit process judgment matrix. The consistency in the recognition of the order of the indicators shows the respondents' unanimous agreement upon which the indicators must be prioritized.

The results for the learning and growth dimension show that C_54_ had the highest weight suggesting that annual professional reeducation of auditors is the most crucial indicator. The average number of years of audit experience (C_52_) and the average age of auditors (C_51_) received considerably low recognition among learning and growth indicators. Per the pairwise comparison results, C_53_ has equal importance with C_55_ and C_56_. Similarly, C_55_ and C_56_ were judged to have equal importance among learning and growth indicators. The results from the CR suggest valid and acceptable judgment (see Table 10).

**Table 10:** Learning and growth indicators.

	Experts/Auditors		Academic Scholars		Audit Commitment Members	
C5	W	Rank	W	Rank	W	Rank
C51	0.063	5	0.080	5	0.070	5
C52	0.049	6	0.060	6	0.052	6
C53	0.199	2	0.268	2	0.235	2
C54	0.335	1	0.280	1	0.319	1
C55	0.182	3	0.174	3	0.168	3
C56	0.173	4	0.137	4	0.157	4
Consistency Checks	6.10 (0.02)		6.16 (0.03)		6.19 (0.03)	

Bolded values represent λmax. CR values are in parenthesis ().

The CR values of all dimensions are all lower than 0.10 indicating that the results are valid and free from logical errors. In line with these results, the weightings of all the indicators are computed (see Table 11). From the perspective of calculating weights, the internal audit process accounts for the highest proportion, indicating that it has the greatest importance and most prioritized criteria in the IAQE index system. In terms of specific indicators, the respondents recognized C_42_ (the proportion of achieving internal audit objectives) as the most priority indicator in the index, followed by C_54_ (average hours of annual professional reeducation of auditors) and C_37_ (audit recommendations are adopted and implemented). C_16_ (number of complaints received by the internal audit department), C_32_ (the rate of difference between internal audit fees and budget), and C_21_ (State-owned Assets Supervision and Administration Commission's control of internal audit functions) were ranked as the least.

**Table 11:** AHP Results of Each of the IAQE indicators.

	Experts/Auditors		Academic Scholars		Audit Commitment Members	
Indicators	W	Rank	W	Rank	W	Rank
C_11_	0.021	18	0.023	16	0.022	17
C_12_	0.009	29	0.010	29	0.009	30
C_13_	0.022	16	0.023	16	0.023	16
C_14_	0.034	12	0.032	12	0.033	12
C_15_	0.014	24	0.013	24	0.015	23
C_16_	0.006	36	0.007	34	0.006	36
C_17_	0.015	23	0.016	23	0.015	23
C_18_	0.009	29	0.010	29	0.009	30
C_21_	0.008	32	0.007	34	0.007	34
C_22_	0.009	29	0.009	31	0.010	29
C_23_	0.012	26	0.013	24	0.012	26
C_24_	0.020	19	0.019	20	0.019	20
C_25_	0.011	27	0.011	27	0.012	26
C_26_	0.022	16	0.022	18	0.022	17
C_27_	0.008	32	0.009	31	0.007	34
C_28_	0.024	15	0.024	15	0.024	14
C_29_	0.008	32	0.008	33	0.009	30
C_31_	0.030	13	0.031	13	0.030	13
C_32_	0.007	35	0.007	34	0.008	33
C_33_	0.018	21	0.018	21	0.019	20
C_34_	0.017	22	0.017	22	0.017	22
C_35_	0.019	20	0.020	19	0.020	19
C_36_	0.052	5	0.052	5	0.051	5
C_37_	0.062	3	0.063	3	0.063	3
C_41_	0.050	6	0.049	6	0.049	6
C_42_	0.120	1	0.116	1	0.118	1
C_43_	0.025	14	0.025	14	0.024	14
C_44_	0.047	7	0.046	7	0.048	7
C_45_	0.044	8	0.044	8	0.043	8
C_46_	0.054	4	0.053	4	0.053	4
C_51_	0.013	25	0.013	24	0.013	25
C_52_	0.010	28	0.011	27	0.011	28
C_53_	0.041	9	0.041	9	0.040	9
C_54_	0.067	2	0.065	2	0.066	2
C_55_	0.037	10	0.039	10	0.038	10
C_56_	0.035	11	0.034	11	0.035	11
Consistency Checks	**15.71** (0.03)		**16.00** (0.04)		**15.93** (0.04)	

Bolded values represent λmax. CR values are in parenthesis ().

### 4.4 IAQE index performance evaluation by listed companies

Following the publications of Li et al. (2020) and Chen et al. (2018), the data was analyzed and the scores for each of the respondent's groups (internal auditors, management, and audit committee members) are presented in Table 12. The total scores for the indicators among the three groups show that all the indicators are high (above 7) indicating that the indicators are useful and the IAQE index is reliable and practicable across multiple companies.

**Table 12:** IAQE index Scores.

			Internal Auditors		Audit Committee Members		Management Members	
Dimension	Indicators	AW	Score	Z	Score	Z	Score	Z
Stakeholder satisfaction (C1)	C_11_	0.022	7.62	0.168	7.72	0.170	7.82	0.172
	C_12_	0.009	7.17	0.067	6.87	0.062	7.21	0.065
	C_13_	0.023	7.65	0.173	7.28	0.167	7.56	0.174
	C_14_	0.033	7.79	0.257	7.65	0.252	7.78	0.257
	C_15_	0.014	7.52	0.105	7.39	0.103	7.58	0.106
	C_16_	0.006	6.73	0.043	7.13	0.043	6.76	0.041
	C_17_	0.015	7.41	0.114	7.57	0.114	7.39	0.111
	C_18_	0.009	7.09	0.066	7.32	0.066	7.19	0.065
Stakeholder contribution (C2)	C_21_	0.007	6.78	0.05	7.11	0.050	6.84	0.048
	C_22_	0.009	6.94	0.065	6.91	0.062	6.93	0.062
	C_23_	0.012	7.22	0.089	7.34	0.088	7.24	0.087
	C_24_	0.019	7.39	0.143	7.46	0.142	7.4	0.141
	C_25_	0.011	7.22	0.082	7.15	0.079	7.14	0.079
	C_26_	0.022	7.45	0.164	6.93	0.152	7.19	0.158
	C_27_	0.008	7.15	0.057	7.22	0.058	7.16	0.057
	C_28_	0.024	7.64	0.183	7.12	0.171	7.35	0.176
	C_29_	0.008	7.61	0.063	7.72	0.062	7.63	0.061
Financial results (C3)	C_31_	0.03	7.37	0.224	7.29	0.219	7.36	0.221
	C_32_	0.007	7.35	0.054	7.62	0.053	7.4	0.052
	C_33_	0.018	7.19	0.132	7.06	0.127	7.14	0.129
	C_34_	0.017	7.11	0.121	6.89	0.117	7.00	0.119
	C_35_	0.02	7.53	0.148	7.36	0.147	7.44	0.149
	C_36_	0.052	7.55	0.39	7.38	0.384	7.46	0.388
	C_37_	0.063	7.96	0.499	7.62	0.480	7.76	0.489
Internal audit process (C4)	C_41_	0.049	7.61	0.375	7.72	0.378	7.63	0.374
	C_42_	0.118	8.84	1.043	7.96	0.939	8.33	0.983
	C_43_	0.025	7.49	0.185	7.56	0.189	7.39	0.185
	C_44_	0.047	7.61	0.358	7.82	0.368	7.65	0.360
	C_45_	0.044	7.87	0.344	7.69	0.338	7.67	0.337
	C_46_	0.053	7.86	0.419	7.69	0.408	7.7	0.408
Learning and growth (C5)	C_51_	0.013	7.09	0.092	6.83	0.089	6.96	0.090
	C_52_	0.011	7.02	0.075	7.26	0.080	7.19	0.079
	C_53_	0.041	7.79	0.317	7.55	0.310	7.62	0.312
	C_54_	0.066	7.93	0.523	7.53	0.497	7.6	0.502
	C_55_	0.038	7.63	0.29	7.45	0.283	7.53	0.286
	C_56_	0.035	7.55	0.262	7.48	0.262	7.65	0.268
Overall score ∑Z				7.739		7.508		7.589

## 5 Discussion

According to the results, 36 indicators were selected for the IAQE index. The indicators were classified into five BSC perspectives based on experts’ opinions and consensus. This consensus is consistent with Chaoli (2008), Nasiripour et al. (2012) Guilong (2013), and Harris and Williams (2020) who support the view that IAQ should be evaluated from varied perspectives. In the IAQE index, eight indicators formed stakeholder satisfaction, nine indicators were selected into stakeholder contributions, seven indicators make up financial results, and six indicators each formed internal audit process and learning and growth perspectives.

All five dimensions were rated to be important for building a comprehensive IAQE index. However, the internal audit process dimension had the highest recognition since the establishment of a scientific and effective internal audit process forms the foundation of the IAQE index. Harrisand Williams(2020) supported this revelation. Thus, topics relating to the internal audit process “proportion of achieving internal audit goals”, “the importance of audit matters”, and “the degree of perfection of internal audit quality assessment results” among others need immediate attention. The experts believe that stakeholder satisfaction and financial results dimensions are the next priority indicators since both indicators directly deal with the quality of internal audit results. Stakeholders' satisfaction with the objectivity of audit evaluation, the accuracy of audit findings, the appropriateness of audit opinions, the operability of audit recommendations is useful in evaluating IAQ. As posited by Souad et al. (2021), stakeholders play key roles in IAQE. As such stakeholder recognition is critical in developing IAQE (Azzali and Mazza, 2018). Financial results directly measure the quality of internal audit results by introducing the main financial performance indicators audited by the internal audit department.

In terms of the analysis of the individual dimensions, the experts revealed priority areas that need immediate attention. “The degree of management's acceptance of the internal audit conclusions” is the most prioritized indicator among the eight stakeholder satisfaction indicators because the indicator reflects the degree to which the company's management approves the results of internal audits. A more effective and higher-quality internal audit work attracts higher management adoption of internal audit conclusions. As revealed by Azzali and Mazza (2018), stakeholders play a key role in IAQE. The experts’ weightings on “Audit committee’s satisfaction with internal audit” and “Management's functional positioning of internal audit” as next priorities areas further support the importance of stakeholder satisfaction in the IAQE index system. The internal audit members composed of independent directors required to have solid financial expertise and rich experience can provide a general understanding of internal audit activities that help improve the quality of the entire enterprise. Additionally, the positioning of internal audit functions by corporate management can guide enterprises to establish internal audit functions and make timely adjustments according to the corporate management requirements of other internal audit departments with more complete audit functions (Gaosong and Leping, 2021). Regarding stakeholder contributions, the “proportion of companies implementing audit recommendation” is the most crucial indicator among stakeholder contribution indicators since the percentage of companies implementing audit recommendations will give higher evaluations to quality audit recommendations and participate in more improvements. Additionally, “management level of reporting on internal audit requirements” and “the audit committee's attention to corporate risks” are priority indicators within stakeholder contributions. Souad et al. (2021) support this finding. This indicator shows how important the audit committee is to corporate risk. If the auditors adapt the appropriate requirements for the audit level of the internal audit department to the internal situation of the enterprise, there is a certain degree of assurance of quality internal audit. Generally, a high level of internal reporting within the organization influences management acceptance of internal audit results.

Financial results indicators established in this study include fraud found internal audit found, findings relating to company’s expenses, adoption and implementation of audit recommendations, and internal audit value added to internal audit cost. HarrisandWilliams(2020) support this finding. However, the AHP analysis revealed that the adoption and implementation of audit recommendations is the most prioritized indicator among financial results indicators. If various departments adopt the audit recommendations and implement them, then there is a greater chance that the results it valid and applicable. Therefore, issues regarding financial reporting in the internal audit may help improve the enterprise’s financial results. In line with this, the experts’ recommended that audit findings and recommendations are key to promoting financial results. The main audit findings not limited to the company’s expenses, production, and marketing cost that have a significant impact on the company's development, operations, and financial status are key priorities. Concerning the internal audit process indicators, the results achieving internal audit goals is the topmost priority. According to the dynamic principle, the internal and external environment of the enterprise, the completion rate of a specific audit plan should not only measure the quality of the process but also pay more attention to the proportion of achieving audit objectives (Gaosong and Leping, 2021). The higher the rate of achieving audit objectives, the higher the effectiveness of internal audits. Hence, improving the internal audit quality process and results received experts’ approval to be given attention within the internal audit process.

Although auditors' education and ability to use information technology received considerably high recognition, the experts recommended professional reeducation of auditors to be given the highest priority and immediate attention among learning and growth indicators. Enterprises are dynamic and keep evolving, hence, through the self-learning process and continuous professional reeducation of internal auditors, there is a higher degree of improving internal audit quality (Gaosong and Leping, 2021). Professional reeducation will expose internal auditors to the latest information and technology needed for quality internal auditing. The high level of consistency among the experts’ judgment depicts their unanimous endorsement of the order of priority in the implementation of the learning and growth indicators. This is because the learning and growth indicators equip internal auditors with professional knowledge and skills that are adaptable to the specific evaluation needs of the enterprises (Zhu et al., 2021).

The IAQE index has several similarities in content with domestic and international literature. At the domestic level, the study is comparable to those of Chaoli (2008), Guilong (2013), and Guizhen (2014). (2019). However, the inclusion of stakeholder and audit committees in the present study provides a wider and more comprehensive view of the IAQE index (Souad et al., 2021; Hazaea et al., 2021). At the international level, this study shares similarities with Ganmei (2015), Azzali and Mazza (2018), Palfi and Bota-Avram (2009) in Indonesia, and Harris and Williams (2020) in the UK. Similarly, the contents of this study share similarities with indicators developed by international bodies ACRA, CAANZ, FRC UK, PCAOB, US CAQ, IOSCO (Federation of European Accountants, 2015). Thus, this study brings together relevant contents from these studies to provide a valid and comprehensive index for evaluating IAQ. The evaluation feedback from the internal auditors and audit committee members of the selected companies provides further support about the relevance of the indicators within the IAQE index system.

## 6 Conclusion and recommendations

The need for a scientific and reliable IAQE index to guide companies to improve internal auditing influenced this study. This study constructed the IAQE index by combining literature review, preliminary analysis, and review of companies’ internal audit guidelines, BSC, Delphi Process, and AHP methodologies. A systematic literature review, review of companies’ internal audit guidelines, BSC, and Delphi approaches resulted in a multilevel IAQE index comprising five dimensions and 36 indicators. Eight indicators were selected stakeholder dimension, nine indicators for stakeholder contribution dimension, seven indicators for financial results dimension, six indicators for internal audit process dimension, and six indicators for learning and growth dimension. The AHP results through experts’ judgment of the dimensions and indicators revealed that all the five dimensions are important in the IAQE index system. Overall, “achieving internal audit objectives” had the highest weight while “the number of complaints received by the internal audit department” had the lowest weight in the IAQE index system. The CR and feedback evaluation from the internal auditors and audit committee members of the selected enterprises revealed the acceptability of the results. In line with internal auditing standards, the developed IAQE index fits well with internal audit components since the index offers an independent, objective assurance and consulting among key stakeholders internal auditing for evaluating and improving the efficacy of risk management, control, and governance systems.

### 6.1 Theoretical implications

Although there has been an attempt to develop an evaluation index for IAQ, this work has a number of theoretical implications. First, unlike earlier research (Chaoli, 2008; Harris and Williams, 2020; Gaosong and Leping, 2021), the IAQE created in this work expands the literature to include stakeholder aspects. As a result, the study provides a comprehensive IAQE index that includes relevant parties in internal audit quality, which is required to design long-term business solutions. Second, relying on the modified BSC approach and Delphi technique, this study decomposed the stakeholder dimensions into stakeholder satisfaction and stakeholder contribution dimensions. This adds unique contributions to the IAQ literature. Third, unlike many studies that used that failed to minimize subjective in the development of their index, this study integrated a systematic literature review, the Balanced Scorecard (BSC), the Delphi approach, and AHP to build the IAQE index and determine the index system's weightings. As a result, the combination of these qualitative and quantitative approaches, as well as ensuring the consistency of expert judgment, reduces subjectivity in the current index. Finally, the AHP results indicate the sequence and priority areas to be followed to attain IAQ. For instance, internal audit dimensions should be prioritized when implementing the IAQE index. By revealing the priority areas, this study helps to minimize the overlaps and subjective preferences identified in the literature (Trotman and Duncan, 2018).

### 6.2 Practical implications

This study offers the following practical contributions. (1) The outcome of the study is relevant to companies seeking to improve their internal audit quality. The robustness of the IAQE index is very important for small and large companies considering the role internal audit quality plays in financial reporting and the survival of a company. Thus, institutional regulators, managers, and audit committees can use this comprehensive IAQE index comprised of multi-dimensions - stakeholder satisfaction, stakeholder contribution, financial results, internal audit process, and learning and growth to strengthen their internal audit quality and financial reporting. (2) Considering the feedback from the listed companies, adopting this index will improve the work efficiency of the executives and their understanding of internal audit activities, and give the full role of corporate governance in the smooth implementation of internal audits. If a company establishes effective internal control, it will ensure the quality of its financial report disclosure and improve the reliability of the information.

### 6.3 Recommendations

Following the study's findings, we make the following recommendations:

1. Companies should use an all-inclusive, reliable, and multilevel IAQE index to guide and evaluate internal audit quality. This will help to embrace internal auditing holistically and resolve potential internal auditing issues. Companies’ evaluation of internal audit quality should broadly focus on stakeholder satisfaction, stakeholder contribution, financial results, internal audit process, and learning and growth dimensions as well as their respective indicators.
2. In evaluating internal audit quality, the companies and key stakeholders ought to prioritize and encourage matters relating to internal audit quality. It is unsuitable to apportion the same level of importance to the key dimensions or take a neutral position when implementing internal audit quality initiatives. Consistent with the study's findings, we suggest that the internal audit process dimension, which focuses on internal audit goals, should be given the highest priority and recognition.
3. Since internal audit quality evaluation relies on the collaboration among the key stakeholders, we recommend smooth collaborations among the stakeholders to ensure that evaluation outcomes and recommendations are adopted and implemented by all the stakeholders. The implementation of an internal audit also requires the close cooperation of multiple departments and personnel.

Although considerable care, companies’ participation, and multiple approaches were followed to develop the IAQE index, we acknowledge some limitations. It is important to state that the survey data used in this data were collected from a specific country, China. Hence, care should be taken in terms of the generalization of the study to other countries. Future research could be conducted to validate the findings in other countries to widen the application of the index.

## Declarations

### Author contribution statement

Ren Kai; Kong Yusheng, PhD; Albert Henry Ntarmah, PhD; Chen Ti, PhD: Conceived and designed the experiments; Performed the experiments; Analyzed and interpreted the data; Contributed reagents, materials, analysis tools or data; Wrote the paper.

### Funding statement

Kong Yusheng was supported by National Natural Science Foundation of China [71973054; 20BGL099; 71371087].

### Data availability statement

Data included in article/supp. material/referenced in article.

### Declaration of interests statement

The authors declare no conflict of interest.

### Additional information

No additional information is available for this paper.

## Figures and Tables

**Figure:**
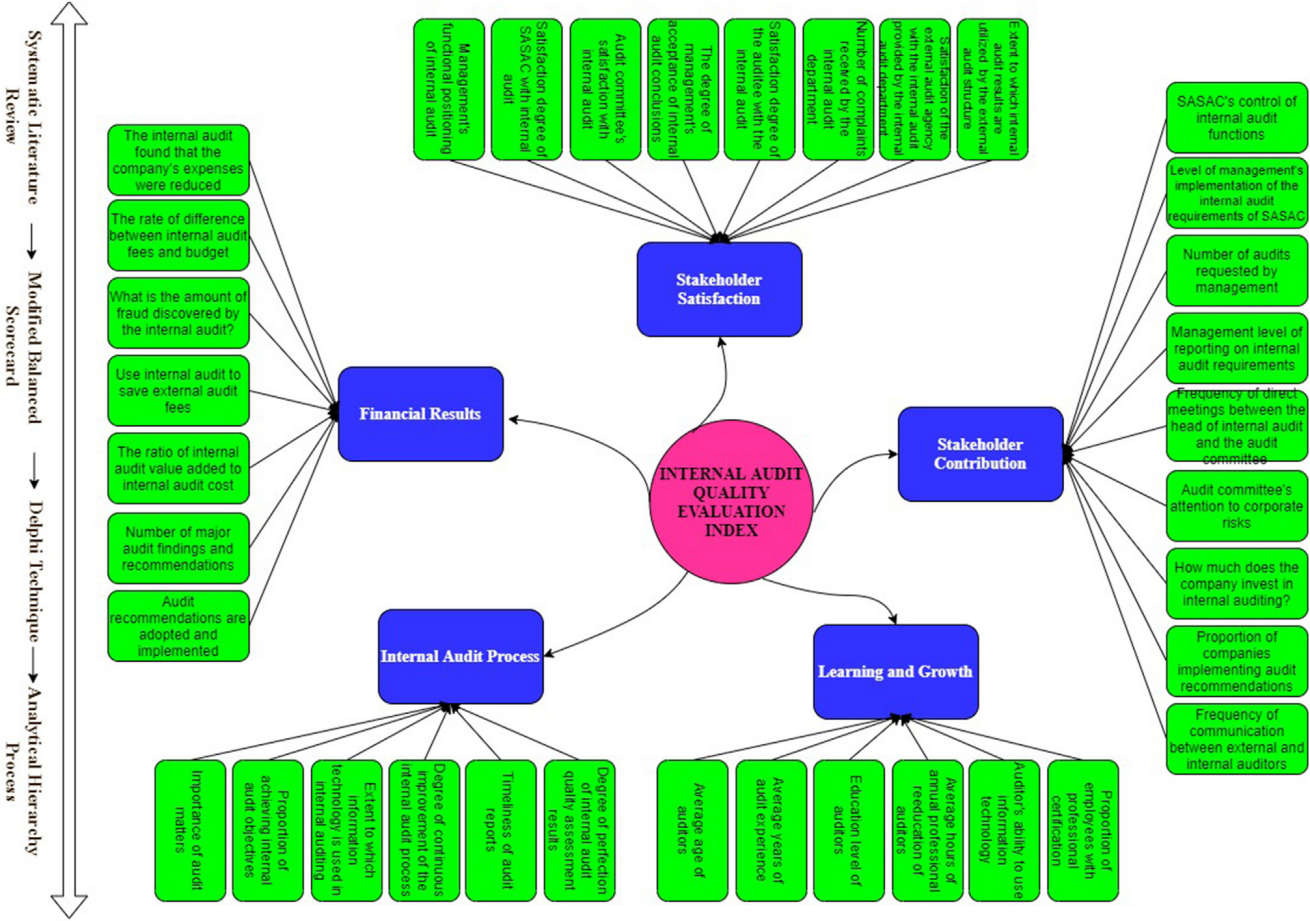


## Appendix

Appendix: Graphical Illustration of Internal Audit Quality Evaluation Index
