# Supplementary material for: Constructing internal audit quality evaluation index: evidence from listed companies in Jiangsu province, China
Source: Heliyon. 2022 Sep 13;8(9):e10598. doi: 10.1016/j.heliyon.2022.e10598 (PMC9508423; doi:10.1016/j.heliyon.2022.e10598)
Supplement: Supplementary File [file mmc1.docx]

**Appendix: Questionnaire**

This survey seeks to evaluate the relevance of the indicators in the internal audit quality evaluation index. We will be pleased if you can help us by responding to the following questions/statements. We assure you that your responses remain confidential.

Please provide your answer by ticking [**√ ]** the appropriate box.

**Section A: Role**

Which of the roles best describes you?

1. Internal auditor [ ]

2. Management [ ]

3. Audit committee members [ ]

**Section B: IAQE Indicators**

Please rate the relevance of each indicator on a 10 points scale ranging from 1 less important to 10 very important.

| **Indicators** | **1** | **2** | **3** | **4** | **5** | **6** | **7** | **8** | **9** | **10** |
| --- | --- | --- | --- | --- | --- | --- | --- | --- | --- | --- |
| 1. ***Stakeholder satisfaction*** |  |  |  |  |  |  |  |  |  |  |
| Management's functional positioning of internal audit |  |  |  |  |  |  |  |  |  |  |
| Satisfaction degree of State-owned Assets Supervision and Administration Commission with internal audit |  |  |  |  |  |  |  |  |  |  |
| Audit committee’s satisfaction with internal audit |  |  |  |  |  |  |  |  |  |  |
| The degree of management's acceptance of internal audit conclusions |  |  |  |  |  |  |  |  |  |  |
| Satisfaction degree of the auditee with the internal audit |  |  |  |  |  |  |  |  |  |  |
| Number of complaints received by the internal audit department |  |  |  |  |  |  |  |  |  |  |
| Satisfaction of the external audit agency with the internal audit provided by the internal audit department |  |  |  |  |  |  |  |  |  |  |
| The extent to which internal audit results are utilized by the external audit structure |  |  |  |  |  |  |  |  |  |  |
| 1. **Stakeholder contribution** |  |  |  |  |  |  |  |  |  |  |
| State-owned Assets Supervision and Administration Commission's control of internal audit functions |  |  |  |  |  |  |  |  |  |  |
| The level of management's implementation of the internal audit requirements of the State-owned Assets Supervision and Administration Commission |  |  |  |  |  |  |  |  |  |  |
| Number of audits requested by management |  |  |  |  |  |  |  |  |  |  |
| Management level of reporting on internal audit requirements |  |  |  |  |  |  |  |  |  |  |
| Frequency of direct meetings between the head of the internal audit and the audit committee |  |  |  |  |  |  |  |  |  |  |
| The audit committee's attention to corporate risks |  |  |  |  |  |  |  |  |  |  |
| How much does the company invest in internal auditing? |  |  |  |  |  |  |  |  |  |  |
| The proportion of companies implementing audit recommendations |  |  |  |  |  |  |  |  |  |  |
| Frequency of communication between external and internal auditors |  |  |  |  |  |  |  |  |  |  |
| 1. **Financial Results** |  |  |  |  |  |  |  |  |  |  |
| The internal audit found that the company's expenses were reduced |  |  |  |  |  |  |  |  |  |  |
| The rate of difference between internal audit fees and budget |  |  |  |  |  |  |  |  |  |  |
| What is the amount of fraud discovered by the internal audit? |  |  |  |  |  |  |  |  |  |  |
| Use internal audit to save external audit fees |  |  |  |  |  |  |  |  |  |  |
| The ratio of internal audit value added to internal audit cost |  |  |  |  |  |  |  |  |  |  |
| Number of major audit findings and recommendations |  |  |  |  |  |  |  |  |  |  |
| Audit recommendations are adopted and implemented |  |  |  |  |  |  |  |  |  |  |
| 1. **Internal audit process** |  |  |  |  |  |  |  |  |  |  |
| Importance of audit matters |  |  |  |  |  |  |  |  |  |  |
| The proportion of achieving internal audit objectives |  |  |  |  |  |  |  |  |  |  |
| The extent to which information technology is used in internal auditing |  |  |  |  |  |  |  |  |  |  |
| The degree of continuous improvement of the internal audit process |  |  |  |  |  |  |  |  |  |  |
| Timeliness of audit reports |  |  |  |  |  |  |  |  |  |  |
| Degree of perfection of internal audit quality assessment results |  |  |  |  |  |  |  |  |  |  |
| 1. **Learning and growth** |  |  |  |  |  |  |  |  |  |  |
| The average age of auditors |  |  |  |  |  |  |  |  |  |  |
| Average years of audit experience |  |  |  |  |  |  |  |  |  |  |
| Education level of auditors |  |  |  |  |  |  |  |  |  |  |
| Average hours of annual professional reeducation of auditors |  |  |  |  |  |  |  |  |  |  |
| Auditor's ability to use information technology |  |  |  |  |  |  |  |  |  |  |
| The proportion of employees with professional certification |  |  |  |  |  |  |  |  |  |  |

Thank you for your time and responses.
